# Men’s perspectives on HIV self-testing in sub-Saharan Africa: a systematic review and meta-synthesis

**DOI:** 10.1186/s12889-020-8184-0

**Published:** 2020-01-15

**Authors:** Mbuzeleni Hlongwa, Tivani Mashamba-Thompson, Sizwe Makhunga, Claudine Muraraneza, Khumbulani Hlongwana

**Affiliations:** 1 Discipline of Public Health Medicine, School of Nursing and Public Health, University of KwaZulu-Natal, Durban, South Africa; 2 CIHR Canadian HIV Trials Network, Vancouver, BC Canada

**Keywords:** HIV testing, HIV self-testing, HIVST, Men, Males, Sub-Saharan Africa

## Abstract

**Background:**

Despite the many HIV testing models implemented in Africa, the level of HIV testing uptake remains relatively poor, especially among men. The HIV self-testing (HIVST) model offers an additional approach for encouraging men to get tested. This study aimed to synthesise evidence on men’s perspectives regarding HIVST in sub-Saharan Africa (SSA).

**Methods:**

The databases searched included PubMed/MEDLINE, American Doctoral Dissertations via EBSCO host; Union Catalogue of Theses and Dissertations; SA ePublications via SABINET Online; World Cat Dissertations; Theses via OCLC; ERIC; CINAH; PsychInfo; Embase, Sociological Abstract, Scopus; and Google Scholar. The World Health Organization (WHO) and The Joint United Nations’ Programme on HIV and AIDS (UNAIDS) websites were further searched. We only extracted qualitative information from the included studies, despite the research method used (qualitative or mixed methods). The Preferred Reporting Items for Systematic Reviews and Meta-Analysis (PRISMA), as well as the Mixed Method Appraisal Tool (MMAT) version 2018, were used to determine the methodological quality of the included studies. NVivo version 11 was used for thematic analysis.

**Results:**

A total of 21,184 articles were identified by the initial search criteria, but only 16 articles were included in the data extraction and quality assessment stage. The following key themes emerged: knowledge of HIVST; acceptability of HIVST; need for HIVST counselling; confidentiality of HIVST; convenience of HIVST; and accuracy of HIVST. The study shows that while HIVST provides men with an alternative, confidential and convenient testing model, the potential for psychological and physical harm remains a challenge.

**Conclusion:**

The introduction of the HIVST strategy has the potential of improving men’s uptake in HIV testing services, thereby contributing towards addressing the first cascade of the 90–90-90 strategy. While HIVST has a potential for addressing men’s barriers to attending clinic settings, such as confidentiality and convenience, it barely addresses the HIVST counselling and accuracy concerns.

## Background

There remains a noticeable gender gap as far as the rates of HIV testing uptake in SSA are concerned, with men remaining harder to reach for HIV testing than their female counterparts [1]. There are many factors that affect men’s uptake of HIV testing services in healthcare clinics, including stigma and confidentiality-related fears [2]. Due to men’s reluctance to visit healthcare facilities [2], there remains a high proportion of HIV positive men who are unaware of their HIV status, some of whom engage in risky sexual intercourse, thereby exposing more women, as well as other men in cases of men who are having sex with other men (MSM), to new HIV infections, which in turn threatens to undermine the progress made towards addressing the HIV epidemic [3]. Men living with HIV are more likely to die early compared to their female counterparts, owing to late diagnosis and antiretroviral therapy (ART) being initiated when the HIV is already at an advanced stage [4–6]. This suggests a particular urgency in regard to designing and implementing innovative strategies to target men for HIV testing services in resource-limited settings in order to address the first cascade of the UNAIDS 90–90-90 programme (90% of all people living with HIV should be diagnosed, 90% of people diagnosed with HIV should be started on ART, and 90% of people started on ART should have a suppressed viral load) [7].

The community and home-based HIV counselling and testing models [8–10] in particular have made some strides towards improving the uptake of HIV testing among men in SSA. However, these strategies become ineffective in highly mobile communities [10]. HIV self-testing (HIVST) offers a new approach to improving men’s HIV testing rates and removing some of the barriers associated with accessing clinic-based HIV testing services by enabling individuals to conduct and interpret their own HIV tests at their own convenient time and in a private space [11–13], whereas HIV self-sampling occurs when individuals collect their own samples (usually blood) and send their specimen to a laboratory for analysis [14]. HIVST can be administered orally (using saliva) or through finger-pricking (drawing of blood). Individuals administer HIVST with little or no training, although written instructions and warnings are provided with the kit. Some of the notable arguments against HIVST involve its high costs, insufficient counselling, and missed opportunities for STI screening [15]. Despite these arguments, HIVST has been shown to be widely acceptable in SSA [11, 12, 16], as well as among key population groups [13]. However, this acceptability has not seemed to have translated into an increase in the uptake of HIVST, especially among men in SSA.

Furthermore, the research evidence regarding men’s perspectives towards HIVST, globally and SSA in particular, remains limited [12]. While more studies are being conducted to develop understanding of HIVST and innovative methods towards hard-to-reach populations globally, similar studies are also necessary for SSA [12]. In SSA, the limited literature on HIVST may be due to the fact that the adoption of HIVST is a recent development. The first phase of the HIVST initiative began in 2015 in SSA and is currently implemented in the following countries: Malawi, Zambia, Zimbabwe, South Africa, Lesotho, and Swaziland. Close to 5 million (4.8 million) HIVST kits in total are expected to be distributed in these countries by the year 2020 [17]. We could not find a similar review which focused solely on qualitative synthesis in SSA to explore men’s perspectives on HIVST using a systematic review and meta-synthesis research method. This study aims to synthesise evidence on men’s perspectives regarding HIVST in SSA.

## Methods

### Design

A systematic search to synthesise qualitative literature for published and unpublished (grey literature) articles was undertaken. Researchers currently working on HIV self-testing in SSA were contacted in an attempt to obtain unpublished articles. The research question aimed to consider studies including qualitative data, but not limited to designs such as phenomenology, grounded theory, ethnography, and action research. Descriptive qualitative studies describing men’s experiences, perspectives, or the effects of the experience of HIVST were considered. Studies including qualitative data were more suitable for this review to explore and synthesise men’s opinions and/or experiences of HIVST. The review protocol was published apriori [18]. The Preferred Reporting Items for Systematic Reviews and Meta-Analyses (PRISMA) guidelines [19] and the Population, Concept, and Context (PCC) framework for determining the eligibility of research question (Table 1) were followed. Since this study utilised a secondary synthesis of data, which is already in the public domain, ethical approvals and consent to participate were not necessary.

**Table 1:** PCC framework

Criteria	Determinants
Population	Men of all age groups in SSA
Concept	HIV self-testing among men
Context	HIV/AIDS

### Identifying the research question

The main research question was: What is the evidence of men’s perspectives on HIVST in SSA?

### Suitability of the question for a systematic review

#### Search strategy

A comprehensive electronic search strategy was conducted in order to identify all relevant grey literature and published studies between January 2005 and February 2019. The search criteria included all studies from this period because studies conducted before 2005 would not reflect key information pertaining to the HIVST model in SSA, mainly due to HIVST being only recently adopted in SSA. However, it was noted that almost all included studies (94%) on HIVST were conducted from 2015 onwards. As part of the search, twelve electronic databases were searched in February 2019: PubMed/MEDLINE, American Doctoral Dissertations via EBSCO host, Union Catalogue of Theses and Dissertations (UCTD); SA ePublications via SABINET Online and World Cat Dissertations; Theses via OCLC; ERIC; CINAH; PsychInfo; Embase, Sociological Abstract, Scopus and Google Scholar. The Medical Research Council (MRC) and Human Sciences Research Council (HSRC) publications, as well as websites from the World Health Organization (WHO) and the Joint United Nations Programme on HIV/AIDS (UNAIDS) were also searched. The reference list of all studies eligible for inclusion were screened for potential additional studies. Boolean terms (AND, OR) and Medical Subject Headings (MeSH) terms formed part of our search strategy. The key search words used were; ‘HIV testing’, ‘HIV self-testing’, ‘HIV self- testing’, ‘Men’, ‘Male’, ‘sub-Saharan Africa’. Sub-Saharan African country names, and truncated terms such as ‘west-Africa’ were also used to ensure that articles indexed using SSA country-specific names or regional terms were retrieved (Appendix). Studies obtained through database searches were exported to Endnote version 7 library for further abstract and full article screening, respectively [20]. The EndNote library “Find full text” option was used to automatically download PDFs of exported studies.

### Study selection and inclusion criteria

The database search was initially conducted against a broad inclusion criterion by the first reviewer (MH). This focused on the title of the articles. All articles identified to be potentially eligible for inclusion in this study were obtained in full texts. Two independent reviewers (MH and SM) then conducted abstracts and full article screenings to identify articles that met all the following inclusion criteria:

- Studies focused on HIVST.
- Articles presented the approach of qualitative data.
- Studies were published between January 2005 and February 2019.
- Articles were conducted in SSA and published in any language, including English.
- Sample either male-only or mixed genders (but with explicit evidence on men).

Those studies published prior to 2005 and those conducted outside of SSA were excluded. Also excluded were studies which did not offer clear and explicit qualitative information on men, despite the research method used.

### Quality appraisal

Methodological rigor in this review was achieved by having two independent reviewers critically appraising the methodological validity of the included studies. A Mixed-Method Appraisal Tool (MMAT), version 2018 [21], was adopted. The MMAT is a critical appraisal tool that has been designed for the appraisal stage of systematic mixed study reviews, like reviews that include qualitative, quantitative, and mixed methods studies (both qualitative and quantitative) (Table 2). The tool helps users to appraise the methodological quality of five categories in studies: (a) qualitative research, (b) randomised controlled trials, (c) non-randomised studies, (d) quantitative descriptive studies, and (e) mixed methods studies. The MMAT tool was used in this study to assess (a) whether each study’s qualitative approach and data collection methods were appropriate to answer the research question; (b) whether the study findings were adequately derived from the data; (c) whether the interpretation of results was sufficiently substantiated by the data; as well as (d) whether there was coherence between qualitative data sources, collection, analysis, and interpretation [21].

**Table 2:** Methodological quality assessment

	Q1	Q2	Q3	Q4	Q5	Q6	Q7	Q8	Q9	Q10	Q11	Q12	Score
Chipungu et al., 2017	y	y	n/a	n/a	n/a	n/a	n/a	y	y	y	y	y	100%
Choko et al., 2011	y	y	n/a	n/a	n/a	n/a	n/a	y	y	y	y	n	86%
Indravudh et al., 2017	y	y	n/a	n/a	n/a	n/a	n/a	y	y	y	y	y	100%
Ritchwood et al., 2019	y	y	n/a	n/a	n/a	n/a	n/a	y	y	y	y	y	100%
Burke et al., 2017	y	y	y	y	y	y	y	n/a	n/a	n/a	n/a	n/a	100/%
Choko et al., 2017	y	y	y	y	y	y	y	n/a	n/a	n/a	n/a	n/a	100%
Conserve et al., 2018	y	y	y	y	y	y	y	n/a	n/a	n/a	n/a	n/a	100%
Conserve et al., 2018	y	y	y	y	y	y	y	n/a	n/a	n/a	n/a	n/a	100%
Harichund et al., 2018	y	y	y	y	y	y	y	n/a	n/a	n/a	n/a	n/a	100%
Jennings et al., 2017	y	y	y	y	y	y	y	n/a	n/a	n/a	n/a	n/a	100%
Kelvin et al., 2016	y	y	y	y	y	y	y	n/a	n/a	n/a	n/a	n/a	100%
Knight et al., 2017	y	y	y	y	y	y	y	n/a	n/a	n/a	n/a	n/a	100%
Makusha et al., 2015	y	y	y	y	y	y	y	n/a	n/a	n/a	n/a	n/a	100%
Martinez Perez et al., 2016	y	y	y	y	y	y	y	n/a	n/a	n/a	n/a	n/a	100%
Matovu et al., 2018	y	y	y	y	y	y	y	n/a	n/a	n/a	n/a	n/a	100%
Ngure et al., 2017	y	y	y	y	y	y	y	n/a	n/a	n/a	n/a	n/a	100%

*Y yes; *N no; *C* can’t tell

**Screening questions (for all types)**

● Q1: Are there clear research questions?

● Q2: Do the collected data allow to address the research questions?

**Qualitative**

● Q3: Is the qualitative approach appropriate to answer the research question?

● Q4: Are the qualitative data collection methods adequate to address the research question?

● Q5: Are the findings adequately derived from the data?

● Q6: Is the interpretation of results sufficiently substantiated by data?

● Q7: Is there coherence between qualitative data sources, collection, analysis and interpretation?

**Mixed methods**

● Q8: Is there an adequate rationale for using a mixed methods design to address the research question?

● Q9: Are the different components of the study effectively integrated to answer the research question?

● Q10: Are the outputs of the integration of qualitative and quantitative components adequately interpreted?

● Q11: Are divergences and inconsistencies between quantitative and qualitative results adequately addressed?

● Q12: Do the different components of the study adhere to the quality criteria of each tradition of the methods involved?

All included articles underwent the initial two screening questions which would indicate whether further methodological quality appraisal was feasible or appropriate. If responses to the initial screening questions were either ‘no’ or ‘can’t tell’ they were excluded from further screening. Qualitative articles were screened with questions three to seven (Table 2). Mixed methods studies were screened with questions eight to twelve. Almost all included studies scored 100%, with just one study scoring 86% [11]. Articles that would score below 50% on the methodological quality assessment were going to be excluded to ensure that included studies had a strong methodological rigor to answer this study’s research question. However, none of the articles were excluded at the methodological quality assessment stage.

### Data extraction

A data collection instrument (using Google Forms) was developed to confirm the study characteristics as well as relevance. The data extraction tool used the following elements: (a) author(s) and date of publication, (b) aim(s) or research questions, (c) primary source data (e.g. quotes from individuals), (d) study population, (e) mean age of participants, (f) gender, (g) percentage of women, (h) percentage of men, (i) geographic setting (rural/urban), (j) study design, (k) type of Intervention and outcomes, (l) most relevant finding, (m) most significant finding, (n) study limitations and implications, as well as (o) interpretations and conclusions from the authors.

### Qualitative synthesis

A thematic synthesis approach that broadly followed the theory outlined by Thomas and Harden (2008) for systematic reviews [22] was used. The theory was developed to address systematic review questions relating to interventions need, appropriateness and acceptability, and effectiveness while ensuring that the key principles developed in systematic reviews were not compromised [22]. Using NVivo version 11 software [23], two independent reviewers (MH and SM) followed the three stages outlined by the thematic synthesis theory: (a) coded the findings of the included studies line-by-line; (b) organised these free codes into related areas to construct descriptive themes; and (c) developed analytical themes [22]. The outcome of coding was verified and discussed with TM-T, a senior researcher and lecturer who also co-authored the manuscript. The process of cross-checking the outcome of coding involved a thorough discussion on the key components of each included article, such as the study aim, setting, number of participants, data analysis method, main findings (themes), limitations, and conclusions.

## Results

**Fig. 1:**
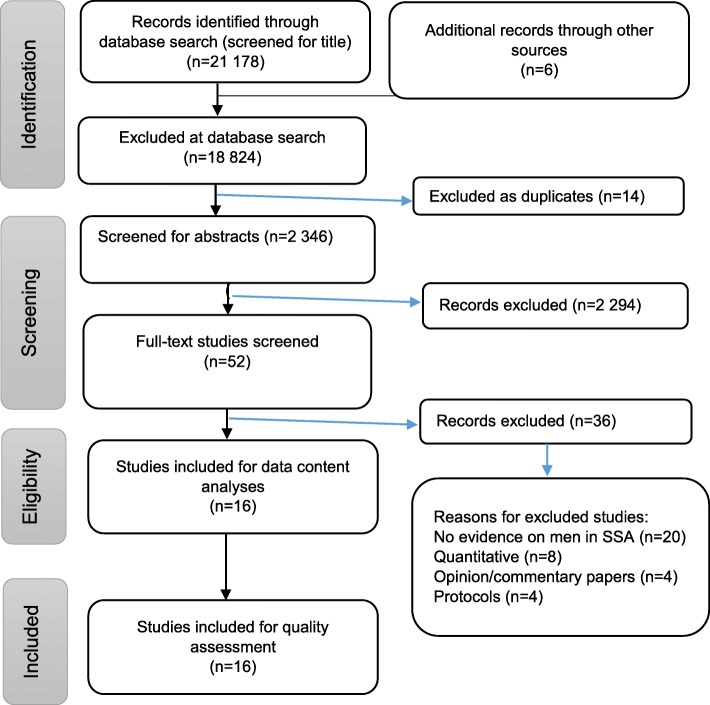
PRISMA flow diagram of the study selection process

The electronic search strategy identified 21,184 references (Fig. 1), which were screened for titles. 18,824 articles were not selected during the database search stage because they did not meet the inclusion criteria. Fourteen duplicates were removed, leaving 2346 articles which were screened for abstracts. A total of 2294 articles were removed at the abstract screening stage because they formed part of the exclusion criteria (i.e. those published prior to 2005, those conducted outside of SSA, and those without qualitative information on men). The researchers further screened 52 full-text articles and excluded 36 of them for the following reasons: eight were quantitative, four were opinion/commentary papers, four were study protocols, and 20 did not present evidence on men in SSA. Therefore, sixteen articles met our inclusion criteria and were included in the quality assessment stage.

### The characteristics of included studies

As a result of the research method used (qualitative or mixed methods), qualitative data from the sixteen included studies were extracted. The included studies were conducted in the following countries: Zambia [24], Malawi [11, 25], South Africa [12, 26–30], Tanzania [31–33], Uganda [34, 35], Zimbabwe and Malawi [36], and Kenya [37]. Twelve studies were predominantly qualitative and four were mixed methods. Quantitative data was not analysed because it was not relevant. Six studies had samples containing males only and ten containing males and females. The samples were predominantly users of HIVST. The search criteria focused on studies published from January 2005 to February 2019, with 94% of the included studies having been published from 2015 onwards. This was expected, given the fact that HIVST is still in the development stage in SSA. The characteristics of the included studies are shown in Table 3.

**Table 3:** Characteristics of included studies

Author & year	Country	Study aim	Sample	Number of participants	Age group	Research Method
Burke et al., 2017	Uganda	To examine values and preferences related to HIVST among community members and health care providers in both mainland and high-risk fishing populations, including with sex workers and fishermen, in Rakai District, Uganda	Males & females	Interviews: 11 – females 10 – males 12 healthcare providersFGDs: 30 – males 25 - females	Not specified	Qualitative (interviews & focus group discussions)
Chipungu et al., 2017	Zambia	To examine the intention to link to care amongst potential HIVST users and the suitability of three linkage to care strategies in Lusaka Province, Zambia	Males & females	Quantitative: 1617 (60% females, 40% males) Qualitative: 64 participants	16–49 years	Mixed methods: Quantitative (cross sectional survey) & qualitative (focus group discussions)
Choko et al., 2011	Malawi	To investigate the potential of supervised oral HIV self-testing in Blantyre, Malawi.	Males & females	Quantitative: 147 – females 136 – males Qualitative: 72 participants (6 groups of 12 participants each)	Median age = 27 years	Mixed methods: Quantitative (cross-sectional) & qualitative (interviews)
Choko et al., 2017	Malawi	To describe the views of pregnant women and their male partners on HIV self-test kits that are woman-delivered, alone or with an additional intervention.	Males & females	31- females 31 – males	Median age for men: 28.5 years; women: 23.5 years	Qualitative (focus group discussions & in-depth interviews)
Conserve et al., 2018	Tanzania	To investigate the reasons and strategies men used to encourage their peers to test for HIV and the outcomes in order to inform the development of a social network-based HIVST intervention for men called STEP (Self-Testing Education and Promotion)	Males	23	Mean age: 27.3 years	Qualitative (interviews)
Conserve et al., 2018	Tanzania	To assess men’s attitudes and personal agency towards HIV self-testing (HIVST) and confirmatory HIV testing in order to inform the development of the Tanzania STEP (Self-Testing Education and Promotion) Project, a peer-based HIV self-testing intervention for young men in Tanzania	Males	23	Mean age: 27.3 years	Qualitative (interviews)
Harichund et al., 2018	South Africa	To assess whether men or women in KwaZulu-Natal displayed a higher acceptance of HIVST and also explored factors that influenced and motivated their acceptability.	Males & females	12 -males; 28 -females	men: 19–37 years; women: 18–37 years	Qualitative (in-depth interviews, Focus group discussions)
Indravudh et al., 2017	Malawi & Zimbabwe	To identify young people’s preferences for HIV self-testing (HIVST) delivery, determines the relative strength of preferences and explores underlying behaviours and perceptions to inform youth-friendly services in southern Africa	Males & females	68 - females 54 – males Qualitative: 8-female interviews (60 FGDs participants) 7 – male interviews (47 FGDs participants)	16–25 years	Mixed methods: Qualitative (interviews & focus group discussions); & experiments
Jennings et al., 2017	Tanzania	To assess perceived costs saved and costs incurred from use of HIVST kits in infrequently- or never-tested Tanzanian men.	Males	23	15 years & older	Qualitative (interviews)
Kelvin et al., 2016	South Africa	To document opinions about self-administered at-home oral HIV testing	Males & females	10 - females 10 - males	18 years & older	Qualitative (interviews)
Knight et al., 2017	South Africa	To assess the perceived usability and acceptability of HIVST among lay users using several self-test prototypes.	Males & females	27 - females; 23 – males	18 years & older	(Qualitative (interviews)
Makusha et al., 2015	South Africa	To explore: interest in HIV self-testing; potential distribution channels for HIV self-tests to target groups; perception of requirements for diagnostic technologies that would be most amenable to HIV self-testing and opinions on barriers and opportunities for HIV-linkage to care after receiving positive test results	Males & females	2: Government Officials; 4: NGOs; 2: Donors; 3 Academic Researchers; 1 Int. stakeholder	18 years & older	Qualitative (in-depth interviews)
Martinez Perez et al., 2016	South Africa	To examine the feasibility and acceptability of unsupervised oral self-testing for home use in an informal settlement of South Africa.	Males	11 - females; 9 - males	18 years & older	Qualitative (couple interviews, in-depth interviews, focus group discussions)
Matovu et al., 2018	Uganda	To explore HIVST perceptions, delivery strategies, and post-test experiences among pregnant women and their male partners in Central Uganda.	Males	17 - females; 15 - males	18 years & older	Qualitative (in-depth interviews)
Ngure et al., 2017	Kenya	To address key questions on feasibility, acceptability and use of HIV self-testing among HIV-uninfected persons initiating PrEP	Males	10 - females; 20 - males	27–38 years	Qualitative (in-depth interviews; focus group discussion) & qualitative
Ritchwood et al., 2019	South Africa	To elucidate concerns and issues regarding HIVST rollout among South African youth	Males & females	Phase 1 (FGDs): 16 females; 19 males, Phase 2a (Observations): 10 females; 10 males Phase 2b (Observations): 20 females; 20 males	18–24 years	Mixed methods: Qualitative (focus group discussions) & observations

### Key themes

The following main themes emerged from the included studies: knowledge of HIVST; acceptability of HIVST; the need for HIVST counselling; confidentiality of HIVST; the convenience of HIVST; and the accuracy of HIVST.

### Knowledge of HIVST

Evidence on knowledge of HIVST was reported on in four articles [12, 31–33]. In two studies conducted in Tanzania, the majority of the men had no prior knowledge of HIVST [31, 32]. Poor knowledge of HIVST was also noted in another study conducted in South Africa [12].

*“I have not heard about it (HIVST). For me, I would not have thought that this is something that you can have access to so easily”* [ [12], p. 3].

Poor knowledge of HIVST did not vary depending on whether a country was implementing HIVST or not. Although this was the case, once the participants became aware of its benefits, they were willing to utilise HIVST after it was introduced to them and promote its use among their peers [12, 31]. Some male participants indicated the need to conduct HIVST awareness campaigns to increase HIVST knowledge and ensure the successful implementation [32, 33], as illustrated by one participant in Tanzania:

*“First, before distributing those test kits there should be a seminar, educating each Tanzanian in general for them to know how to use the kit and then getting the results and what to do after that, this will make someone more aware”* [ [32], p. 9].

Such campaigns may be conducted at community levels as well as in person, at clinics or via pamphlets, or through media channels such as television, radio, and newspapers [32].

### Acceptability of HIVST

The evidence on acceptability was reported on in nine studies [11, 12, 25, 26, 31–34, 37]. The majority of men showed a willingness to use HIVST in studies conducted in Malawi [11], Tanzania [32, 33], South Africa [26], and Kenya [37], with most of the participants having administered HIVST in the past in Tanzania [33]. The oral HIVST was preferred over finger-pricking [26], although respondents agreed that HIVST, in general, was easy to use [26, 37]. One male participant in South Africa stated:

*“It was easy and good. Well, initially I was a bit nervous because I was just starting. But now I’m getting more comfortable because I’ve seen how it works”* [ [26], p. 4].

The acceptability of HIVST was not influenced by participants’ prior knowledge nor awareness of HIVST [12, 32] and did not significantly vary by age, marital status, level of education, or socio-economic status [11]. Some men further indicated that they would recommend HIVST to friends and family members [11]. Others thought that their friends who generally resisted HIV testing would be encouraged to try the HIVST model [31], especially because this HIV testing model could be perceived as addressing the barriers associated with the clinic-based HIV testing model [25], as expressed by one male participant in South Africa:

*“I would rather not go to the clinic once I know how to use it I can then test myself”* [ [26], p. 4].

Also, using women who attend ANC clinics to deliver HIVST to their male partners has been seen to be acceptable by men in Uganda and Malawi, and it is perceived to improve partner and couples’ HIV testing [25, 34].

*“I feel like it’s acceptable because maybe the day that the woman wants to go to ANC clinic you might not be able to escort her, so she can just bring you the test kits when she is coming back from ANC and the next time she is going for ANC then you can go together”* [ [25], p. 4].

### Need for HIVST counselling

The evidence on the importance of counselling prior to using HIVST was reported in eleven articles [12, 24–32, 35]. Studies conducted in Zambia, South Africa, Tanzania, and Malawi indicated that conducting pre-test and post-test counselling is crucial to address potential psychological and physical harm because people are likely to react differently to HIV positive test results. Some may become suicidal or initiate verbal or physical partner violence due to their fear and/or poor knowledge of how to manage the HIV positive result [24, 25, 29, 30, 32]. Below are some comments made by male participants in Zambia and South Africa:

*“If a person is positive, they need to find people who can help [them], so that they can be comforted and not have the feeling of saying ‘why have I been found positive or what can I do?’.*. *. Others commit suicide and they tell themselves they are better off dying than suffering with the illness. So, they should be counselled so that they can understand”* [ [24], p. 8].

*“I will just take the rope and hang myself because I know there is no one who will shout at me. Even if I’m crying, no one will comfort me, but here are things [ARVs] to prevent [death] and even the government is bringing the pills and you can prevent [death] with them … because the disease doesn’t kill you if you can go to the counsellors, the counsellor will tell you that okay, alright, you have the disease and the disease doesn’t kill you”* [ [29]*,* p. 8].

Studies conducted in Malawi, Tanzania, Uganda, and South Africa also supported the importance of pre- and post- HIV counselling for men to ensure that men are not left isolated [11, 12, 26, 28, 29, 31, 35]. The pre-HIVST counselling would likely assess men’s readiness to respond to a potential HIV positive test outcome [32]. Some men, as noted in the comment made by males in Tanzania, indicated that they would educate their peers to take necessary steps, like seeing a healthcare worker for confirmatory results, as well as starting ART treatment [31]:

*“If it will not be possible to check pressure then there is another way where you look at how the person is... Through conversation with the client you are needed to look at him and say that from my questions and his responses is he not going to commit suicide if we give him this instrument”* [ [32], p. 4].

*“I will advise him by telling him that it’s alright you have tested yourself alone but you cannot stay alone you must go to the doctors to be counselled”* [ [31], p. 1192].

While men acknowledged the importance of pre-HIVST counselling, most did not desire the face-to-face counselling with healthcare providers [11, 12], but appreciated alternative forms of counselling that would maintain their privacy, such as sharing information via mobile test messaging services, paper-based counselling information, or phone calls [26]. Despite these preferred methods of counselling, one male from the non-governmental organisation (NGO) sector in South Africa emphasised that HIVST poses challenges as far as counselling is concerned and that there should not be any HIV testing without counselling, as this is an important component for HIV self-testers:

*“But most important... we need to ensure there will be constant access to counselling. We should not undermine the value of counselling. That is why whenever we talk about HIV testing, or whatever the case might be, we talk HIV counselling and testing...You will never find HIV testing that does not have the C, so the C for me is a crucial part”* [ [27], p. 5].

### Confidentiality of HIVST

The HIV self testing model and confidentiality were discussed in eight articles [12, 25–28, 31, 32, 37]. Due to a perceived lack of privacy, confidentiality, and the stigma in healthcare facilities, men believed that the HIVST presented a feasible alternative for them to test for HIV [12, 25, 26, 31, 32, 37]. Privacy and confidentiality were primary reasons men preferred HIVST [31]*,* as noted by a male participant in a study conducted in Tanzania:

*“It will give people privacy as you can take it and go test anywhere in privacy as most of the time people fear going to health clinics as they may meet someone they know or they know a worker there who after testing might go spread the results”* [ [31], p. 1191].

In addition to that, some men preferred HIVST so that they could know their own HIV test results first, especially if they have had multiple sexual partners, as revealed by a male participant in Malawi:

“*… you just go out secretly and follow the method and right there it’s as easy as drinking a glass of water. You quickly place it in the bottle and hide it since you want to check yourself first (participants laugh). When the results are out you will check them, and you will know the outcome yourself right?”* [ [25], p. 4].

While HIVST seems to be beneficial for men, a study conducted in South Africa cautioned that there still remains challenges where men have to collect HIVST kits at healthcare facilities when this programme is up-scaled. The suggestion was that it was important to establish distribution points, especially to encourage men to utilise the programme [12].

### Convenience of HIVST

Evidence of the convenience of HIVST was reported in nine studies [12, 26, 29, 32–37]. Specifically, men preferred HIVST because it was viewed as efficient and convenient [26, 29, 32, 33, 37]. It was also a non-disruptive testing option [33], especially important for those men who, due to long working hours, had difficulty attending clinic settings [12, 32–34, 36]. A clinic setting was also perceived as being time-consuming, as a result of long queues, waiting times [12], and travelling [33]. As noted by male participants in South Africa, Kenya, and Uganda:

*“It’s convenient, you don’t have to go to the clinic and be in a long line, and end up not getting tested in the end, because they work within certain time frames for certain services”* [ [12], p. 4].

*“I think it is right because sometimes there are queues at clinics. And also I am afraid that people will see me in that queue and know that I came for HIV test whereas at home it is easy and everything you do is your secret”* [ [26], p.4].

*“With HIV self-testing you do not require any bus fare, anytime you can just test yourself”* [ [37], p.6].

Therefore, the convenience of HIVST stems from the fact that men can easily use HIVST kits at their own convenience, as well as in the comfort of their homes [34]. This addresses the gap posed by a lack of time to attend clinic settings [34].

### Accuracy of HIVST

The accuracy of HIVST was discussed in five articles [30, 32–34, 37]. Due to the fact that HIVST requires a confirmatory HIV test, this reduces trust in the HIVST results [32], as some men remained sceptical whether or not the HIVST can really test for HIV, especially in instances where saliva is used (oral) instead of a blood sample:

*“I asked myself ‘is this really true’? Can really a person just get that ‘spoon’ [the kit] and pass it on the gum and then*. *.*. *[he spreads his hands] and test for HIV*?” [ [34]. p.4].

Due to doubts about the accuracy of HIVST, some men believed that a clinic setting was actually a better choice for HIV testing because they do not believe that HIVST results are accurate.

*“How sure are you about this kit? I don’t trust this kit. Why should I have to go back to the clinic and get tested again after using [the HIVST kit] and [to potentially] test positive? I cannot use [the HIVST]. I’d rather go to the clinic and use blood test, not [the blood HIVST]”* [ [30], p.6].

Men who are not well-educated were perceived to have a high risk of misinterpreting or getting inaccurate results due to their poor capacity to administer the HIVST kit [32, 33], this was illustrated by a male participant from Tanzania:

*“That is the challenge which I will get as I will not be confident as if I will go to the hospital … I mean I will not be more confident that the instrument has shown correctly the results.*. *. Perhaps there may be a certain mistake which I have made or there may be something which I have done wrong … Because I may do it wrong and it shows me that I am HIV negative while I am HIV positive, so don’t trust myself”* [ [32], p.8].

However, men’s attitudes towards seeking an HIV confirmatory test based on the results of HIVST was high:

“*Truly if I see two lines have appeared I will be ready to move from this place to the responsible place to verify my results” [* [32]*, p.8].*

Although very low in numbers, some men indicated that they would not seek HIV confirmatory test after the HIVST and linkage to ART treatment due to confidentiality and stigma concerns posed by attending the clinic setting [32].

## Discussion

This study revealed evidence regarding men’s perspectives on HIVST in SSA. The results show that men’s knowledge of HIVST remains low in SSA, despite the high acceptability shown when the HIVST was introduced. The study also found that while HIVST provided men with alternative testing model, given its confidentiality and convenience, there remained issues as far as pre- and post-counselling, given the potential psychological and physical harm resulting from different reactions to HIV positive test results. Due to their low confidence in administering HIVST and the use of saliva to test for HIV (oral HIV test), some men do not trust the accuracy of HIVST results. This suggests the importance of educating men about the HIVST model.

While several studies have documented the high acceptance of HIVST among populations [16, 38–40] and its convenience [41, 42], this, to our knowledge, is the first systematic review and meta-synthesis to assess men’s perspectives on HIVST in SSA. While education is key to changing men’s perspectives and improving HIV knowledge, this study further revealed that HIV self-testing is critical to addressing issues of confidentiality and convenience [9, 16, 43, 44]. Therefore, this strategy is key to supporting the UNAIDS plans aimed at addressing masculinity issues, one of which is men’s engagement in HIV testing [45].

The findings of this study, consistent with the findings of other studies conducted in resource-limited settings, revealed that, given the many barriers associated with HIV testing in a clinic setting, more men preferred to engage themselves with HIV testing services that were conducted outside this setting [16, 46–51]. In addition to that, the HIVST model further empowers young people and sexually active individuals to be independent and have the option to choose the location and timing of the test and to control the disclosure of their results [36]. This review further revealed that the oral HIVST model is acceptable among men, supporting the findings of other studies conducted among other study populations in different settings [11, 52]. Like other studies in different settings, this study also found similar concerns regarding the need for HIVST counselling [53, 54], as well as the potential for incorrect interpretation of HIV self-testing results [55]. Notwithstanding the concerns that men raised, the findings support the theory that the HIVST model has the potential to increase the uptake of HIV testing, especially among men. However, the concerns mentioned above need to be addressed.

### Strengths and limitations

Although the searches included a wide range of databases, the overall search strategy may have been biased towards public health and social sciences. While the review included any article published in any language, our search was conducted using only English terms. Nevertheless, we believe that the search strategy was comprehensive in reviewing public health and social sciences literature on men’s perspectives on HIVST in SSA. All included studies underwent quality appraisal using an approved tool, the MMAT. This review also included studies conducted in countries in SSA that are not implementing the HIVST initiative, however, these are not likely to have influenced our findings because similar patterns of men’s perspectives on HIVST were observed across all the included studies conducted in different countries. Given that only a handful of studies met our inclusion criteria, the findings of this review may not be generalised across all men in SSA. However, understanding men’s perceptions on HIVST is important.

### Recommendations for future research

The study findings show that the HIVST has the potential to improve men’s uptake of HIV testing in SSA [56] as it has been shown to attract more men in similar settings [11, 57–59]. The research on HIVST is still in its infancy stages, especially in the SSA region. This is a reason why only a few studies met the inclusion criteria, which focussed specifically on men. Therefore, it is recommended that more studies should be conducted among men regarding HIVST. The focus on men’s perspectives on HIVST is important to understand the challenges and opportunities as far as HIVST implementation is concerned.

### Implications for practice

This study revealed that there is high acceptance of HIVST among men in SSA, although prior knowledge of HIVST was not widespread. This suggests the importance of implementing community-level campaigns aimed at educating men about HIVST, given the potential of this HIV testing strategy to attract hard-to-reach men. When the implementation of HIVST is up-scaled, the implementers should develop strategies aimed at ensuring that all potential HIVST users are counselled and supported to assess their readiness to use HIVST, as well as the potential psychological and physical risks associated with a positive HIV test result.

## Conclusion

The introduction of the HIVST model is important for improving men’s uptake of HIV testing services. While HIVST addresses men’s barriers, such as confidentiality and convenience regarding attending clinic settings, there remains a gap in terms of HIVST counselling and accuracy, with the knowledge remaining poor among potential users. There also remains a gap in research to address the risks associated with HIVST testing strategies versus the potential benefits, especially among men in SSA.

## Data Availability

All the data analysed and reported in this paper were from published literature, which is already in the public domain.

## Appendix

**Table 4:** Sub-Saharan African search filter

PubMed	
(“hiv”[MeSH Terms] OR “hiv”[All Fields]) AND testing [All Fields]) OR ((“hiv”[MeSH Terms] OR “hiv”[All Fields]) AND self-testing [All Fields])) OR ((“hiv”[MeSH Terms] OR “hiv”[All Fields]) AND (“ego”[MeSH Terms] OR “ego”[All Fields] OR “self”[All Fields]) AND testing [All Fields])) AND (“men”[MeSH Terms] OR “men”[All Fields])) OR (“male”[MeSH Terms] OR “male”[All Fields])) AND (“africa”[MeSH Terms] OR “africa”[All Fields])) OR “Sub-Saharan Africa”[All Fields]) OR (“angola”[MeSH Terms] OR “angola”[All Fields])) OR (“benin”[MeSH Terms] OR “benin”[All Fields])) OR (“botswana”[MeSH Terms] OR “botswana”[All Fields])) OR (“burkina faso”[MeSH Terms] OR (“burkina”[All Fields] AND “faso”[All Fields]) OR “burkina faso”[All Fields])) OR (“burundi”[MeSH Terms] OR “burundi”[All Fields])) OR (“cameroon”[MeSH Terms] OR “cameroon”[All Fields])) OR (“cabo verde”[MeSH Terms] OR (“cabo”[All Fields] AND “verde”[All Fields]) OR “cabo verde”[All Fields] OR (“cape”[All Fields] AND “verde”[All Fields]) OR “cape verde”[All Fields])) OR “Central African Republic”[All Fields]) OR (“chad”[MeSH Terms] OR “chad”[All Fields])) OR (“comoros”[MeSH Terms] OR “comoros”[All Fields])) OR “Congo brazzaville”[All Fields]) OR “Democratic republic of congo”[All Fields]) OR “Cote d’Ivoire”[All Fields]) OR (“djibouti”[MeSH Terms] OR “djibouti”[All Fields])) OR “Equatorial Guinea”[All Fields]) OR (“eritrea”[MeSH Terms] OR “eritrea”[All Fields])) OR (“ethiopia”[MeSH Terms] OR “ethiopia”[All Fields])) OR (“gabon”[MeSH Terms] OR “gabon”[All Fields])) OR (“gambia”[MeSH Terms] OR “gambia”[All Fields])) OR (“ghana”[MeSH Terms] OR “ghana”[All Fields])) OR (“guinea”[MeSH Terms] OR “guinea”[All Fields])) OR “Guinea-Bissau”[All Fields]) OR (“kenya”[MeSH Terms] OR “kenya”[All Fields])) OR (“lesotho”[MeSH Terms] OR “lesotho”[All Fields])) OR (“liberia”[MeSH Terms] OR “liberia”[All Fields])) OR (“madagascar”[MeSH Terms] OR “madagascar”[All Fields])) OR (“malawi”[MeSH Terms] OR “malawi”[All Fields])) OR (“mali”[MeSH Terms] OR “mali”[All Fields])) OR (“mauritania”[MeSH Terms] OR “mauritania”[All Fields])) OR (“mauritius”[MeSH Terms] OR “mauritius”[All Fields])) OR (“mozambique”[MeSH Terms] OR “mozambique”[All Fields])) OR (“namibia”[MeSH Terms] OR “namibia”[All Fields])) OR (“niger”[MeSH Terms] OR “niger”[All Fields])) OR (“nigeria”[MeSH Terms] OR “nigeria”[All Fields])) OR (“reunion”[MeSH Terms] OR “reunion”[All Fields])) OR (“rwanda”[MeSH Terms] OR “rwanda”[All Fields])) OR “Sao Tome and Principe”[All Fields]) OR (“senegal”[MeSH Terms] OR “senegal”[All Fields])) OR (“seychelles”[MeSH Terms] OR “seychelles”[All Fields])) OR (“sierra leone”[MeSH Terms] OR (“sierra”[All Fields] AND “leone”[All Fields]) OR “sierra leone”[All Fields])) OR (“somalia”[MeSH Terms] OR “somalia”[All Fields])) OR “South Africa”[All Fields]) OR (“sudan”[MeSH Terms] OR “sudan”[All Fields])) OR (“swaziland”[MeSH Terms] OR “swaziland”[All Fields])) OR (“tanzania”[MeSH Terms] OR “tanzania”[All Fields])) OR (“togo”[MeSH Terms] OR “togo”[All Fields])) OR (“uganda”[MeSH Terms] OR “uganda”[All Fields])) OR “Western Sahara”[All Fields]) OR (“zambia”[MeSH Terms] OR “zambia”[All Fields])) OR (“zimbabwe”[MeSH Terms] OR “zimbabwe”[All Fields])) OR “west africa”[All Fields]) OR “east africa”[All Fields]) OR “southern africa”[All Fields])	

## Abbreviations

HIVST: HIV self-testing
MeSH: Medical Subject Headings
MMAT: The Mixed Method Appraisal Tool
PCC: Population, Concept, and Context
PRISMA: We used the Preferred Reporting Items for Systematic Reviews and Meta-Analysis
SSA: Sub-Saharan Africa
UCTD: Union Catalogue of Theses and Dissertations
UNAIDS: The AIDS and AIDS
WHO: World Health Organisation
